# Effects of Nonsurgical Periodontal Therapy on Clinical Response, Microbiological Profile, and Glycemic Control in Malaysian Subjects with Type 1 Diabetes

**DOI:** 10.1155/2014/232535

**Published:** 2014-07-23

**Authors:** Samira Mukhtar Buzinin, Aied Mohammed Alabsi, Alexander Tong Boon Tan, Vui King Vincent-Chong, Dasan Swaminathan

**Affiliations:** ^1^Department of Restorative Dentistry, Faculty of Dentistry, University of Malaya, 50603 Kuala Lumpur, Malaysia; ^2^Oral Cancer Research and Coordinating Center, Department of Oral Biology and Biomedical Science, Faculty of Dentistry, University of Malaya, 50603 Kuala Lumpur, Malaysia; ^3^Department of Endocrinology, University of Malaya Medical Center, 50603 Kuala Lumpur, Malaysia; ^4^Oral Cancer Research and Coordinating Center, Department of Oro-Maxillofacial and Medical Science, Faculty of Dentistry, University of Malaya, 50603 Kuala Lumpur, Malaysia

## Abstract

The association between diabetes mellitus and chronic periodontal disease has long been established. Most of the researches linking these two very common chronic diseases were based on type 2 diabetes mellitus and chronic periodontal disease. However, this study was conducted to investigate the association between type 1 diabetes and chronic periodontal disease in Malaysian subjects. Forty-one Malaysian subjects, of which 20 subjects were type 1 diabetics and with chronic periodontal disease (test group) and 21 subjects with only chronic periodontal disease (control group), were included in the study. Periodontal parameters and plaque samples for microbiological evaluation were done at baseline, 2 and 3 months after nonsurgical periodontal therapy. Blood samples were taken from only the test group and evaluated for HbA1c at baseline and 3 months after periodontal therapy. There were no statistically significant difference in periodontal parameters between groups (*P>0.05*) and no significant improvement in the level of HbA1c in the test group. Microbiological studies indicated that there were significant reductions in the levels of the tested pathogens in both groups. The results of our study were similar to the findings of several other studies that had been done previously.

## 1. Introduction

Type 1 diabetes represents approximately 5 to 10% of all diagnostic cases of diabetes. It is characterized by chronic hyperglycemia caused by autoimmune pancreatic *β*-cells destruction generally leading to total loss of insulin secretion [1]. Periodontal disease is a chronic inflammation that affects the tissues surrounding the teeth in response to accumulation of bacterial biofilm on the teeth [2]. It has been established that bacterial pathogens and their products play an essential role in the initiation of the chronic inflammatory process causing damage to periodontal tissues. The host response appears to play a key role in pathogenesis of periodontitis by amplifying the destructive inflammatory process initiated by the bacterial insult [3]. The complex pathogenesis of this disease is further complicated by the coexistence of systemic diseases, such as diabetes, which has the potential to aggravate the manifestations of periodontitis [4]. Periodontal disease and diabetes mellitus belong to a pathologic condition in which both diseases could negatively interfere with each other, constituting a bidirectional relationship with diabetes increasing the risk for periodontitis and periodontal inflammation negatively affecting glycemic control and the progression of vascular complications. Diabetic individuals, both type l and type 2, experience a higher incidence of periodontitis and the severity of the disease correlates with the duration of diabetes and glycemic control reflected by glycated hemoglobin (HbAlc) levels in the blood [5]. Type l diabetes has been recognized as an important modifier of periodontal disease [6, 7]. Numerous factors related to diabetes have been proposed to increase the severity of periodontal disease in diabetic individuals, such as vascular abnormalities, neutrophil dysfunction, nonenzymatic glycosylation, altered collagen metabolism, and altered monocytic response [8].

Conversely, periodontal disease may be a critical factor for worsening glucose intolerance among patients with diabetes [9] and may increase the risk of diabetic complications. Periodontitis may initiate or propagate insulin resistance by enhancing activation of the overall systemic immune response initiated by cytokines [10]. Elevated circulating levels of tumor necrosis factor-*α* (TNF-*α*), interleukin-6 (IL-6), and high-sensitivity capsular reactive protein (hs-CRP), which can impair insulin resistance and thereby reduce glycemic control, have been shown in a previous study [11]. This way, the control of periodontal disease is necessary for better systemic health in these individuals.

Numerous studies have been published on the effect of periodontal therapy on glycemic control and some of these researchers have found beneficial effect on glycemic control [12–15]. Other studies have demonstrated no significant effect of periodontal therapy on metabolic control [9, 16, 17]. Due to the contradicting findings in the literature, we wanted to evaluate the influence of nonsurgical periodontal therapy on the metabolic control in type 1 diabetes in Malaysian subjects. We also compared the effect of nonsurgical periodontal therapy on clinical parameters of periodontal disease between subjects with type 1 diabetes and nondiabetic subjects and the effect of periodontal therapy on quantity of periodontal pathogens of these subjects.

## 2. Material and Methods

### 2.1. Sample Size

Sample size was calculated based on expected mean difference in the reduction of HbA1c in diabetic group of around 0.9–1.0% [8]; it was calculated that at least 15 patients would be needed to detect this difference with 80% power and two sided type 1 error of 5%. The basic formula used was

(1) $$\begin{array}{c} m=\frac{2*{\left[Z_{\left(1-\alpha /2\right)}+Z_{\left(1-\beta \right)}\right]}^{2}}{\Delta },\\ Z_{\left(1-\alpha /2\right)}=\text{significance}\;\text{level},\\ Z_{\left(1-\beta \right)}=\text{power},\\ \Delta =\frac{\text{standard}\;\text{difference}}{\text{effective}\;\text{size}}. \end{array}$$

### 2.2. Study Population

Forty-one of which 20 subjects were type 1 diabetics and with chronic periodontal disease and 21 subjects with only chronic periodontal disease were enrolled into this study. Patients diagnosed with moderate to advanced periodontitis, established from their periodontal parameter status and dental panoramic radiological findings, and agreeable patients who fulfilled the inclusion criteria were included in this study. Ethical clearance for this study was approved by both Faculty of Dentistry ethical committee (reference number DF OP 1304/0019(P)) and Faculty of Medicine ethical committee (reference number 962.22) at the University of Malaya for conducting this clinical study on human individuals. The recruited patients were brought to the Postgraduate Clinic, Faculty of Dentistry, for clinical examination. All subjects were briefed on the aims and method of the study in detail through verbal explanation and patient information sheets were given to all subjects at the beginning of the study. Consent was obtained from all recruited subjects. In the inclusion criteria, all subjects who participated in this study had to have a minimum of 12 natural teeth present and should have been diagnosed with periodontal disease with 5 or more sites of pockets of ≥5 mm and probing attachment level (PAL) ≥3 mm [17]. The age of the subjects was between 20 and 65 years of either gender and subjects of the test group should have been diagnosed with type 1 diabetes mellitus [18]. The following exclusion criteria were considered: subjects who were pregnant or smokers, subjects who had received periodontal treatment within the last 6 months [13, 15], and subjects with any history of antibiotics within the last 3 months [13, 15] and nonsteroidal anti-inflammatory drug (NSAIDS) use within the last 3 months [9].

### 2.3. Experimental Design

Examination of periodontal parameters was done for all subjects of both groups at baseline, 2 and 3 months after treatment (Table 2). For the test group, blood samples were collected at baseline and at the end of three months after periodontal treatment for measuring the glycated hemoglobin (HbA1c). Plaque samples were collected from all subjects at baseline and 3 months after periodontal therapy. After recording the periodontal variables, subjects in both groups received oral hygiene instructions and underwent full mouth debridement in a single session using manual instruments (Graceys curette) combined with ultrasonic scaling.

All subjects were recalled at the end of second and third months after treatment and, at each time, the anamnesis was updated and questioned about any changes in medications related to diabetes therapy and alterations in lifestyle.

### 2.4. Periodontal Examination

After selection of subjects for the study, their dental and medical histories were taken. The subjects were then administered a periodontal clinical examination performed in six sites per tooth (excluding third molars) by a single trained calibrated examiner. The periodontal parameters that were assessed were visible plaque index (VPI) and gingival bleeding index (GBI) [19]. Probing pocket depths (PPD) and probing attachment level (PAL) were evaluated using a Florida probe. Orthopantograms were taken for radiographic assessment of alveolar bone resorption for all the subjects.

### 2.5. Metabolic Measurement

Blood samples were collected from all subjects in the test group at baseline before treatment and at the end of 3 months after treatment at the University of Malaya Medical Centre and sent to the diagnostic laboratory of the Medical Centre for measurement of the concentration level of (HbA1c) (%), which was measured by high performance liquid chromatography. Standardization of this procedure was achieved by sending all blood specimens to the same diagnostic laboratory.

### 2.6. Sample Collection and Bacterial DNA Isolation

After all supra gingival plaque and calculus were removed using sterile Gracey's curette, sampling sites were isolated using cotton roll. The selected teeth were then air dried and sterile paper point (size 40) was inserted into deepest pocket of each quadrant for 30 seconds. The paper points, 4 in total for each subject, were then packed into microcentrifuge tube containing 1.5 mL of phosphate buffer solution (PBS). The samples were stored at −20°C until being ready for DNA extraction.

Samples were thawed and vortexed for 10 seconds. After removing the paper points from the tubes, samples were then pelleted by refrigerated centrifuge at 32,000 rpm for 10 minutes at 4°C. The pellet was used directly for total bacterial genomic DNA extraction using QIAamp DNA Mini Blood Mini Kit (Qiagen, GmBH Germany) according to the manufacturer's instructions. The quality (A260/A280, A260/230) and concentration of the gDNA (ng/*μ*L) were determined using the Nanodrop spectrophotometer ND-2000 (NanoDrop Technologies, Wilmington, DE, USA).

### 2.7. Real Time PCR Detection

The standard curves of* P. gingivalis* (PG),* A. actinomycetemcomitans* (AA), and* T*.* forsythia* (TF) were carried out in 6 series of 10-fold dilutions from 2 × 10^5^ to 2 × 10^0^ according to the manufacturer's instruction (PrimerDesign genesig Kit, Southampton, United Kingdom). The gDNA of each sample was obtained and the quantification of PG, TF, and AA was performed using 7500 Fast Real Time PCR System (Applied Biosystems, Foster City, CA, USA) according to the manufacturer's protocol (PrimerDesign genesig Kit, Southampton, United Kingdom). The reaction mixture for the qPCR was done in a total volume of 20 *μ*L consisting of 5 *μ*L of genomic DNA (5 ng/*μ*L), 10 *μ*L of oasig 2X qPCR Mastermix (PrimerDesign genesig Kit, Southampton, United Kingdom), 1 *μ*L of primer/probe mixed assay (FAM reporter), and 4 *μ*L of nuclease free water. Quantitative PCR was performed on an ABI 7500 Fast Real Time PCR System (Applied Biosystems, Foster City, CA, USA) using the suggested manufacturer's PCR conditions as follows: initial denaturation at 95°C for 15 minutes followed by 50 cycles of denaturation for 10 seconds at 95°C and annealing for 60 seconds at 60°C. The reporter dye (FAM) signal was measured relative to the reference dye (ROX).

### 2.8. Statistical Analysis

SPSS version 18 (SPSS Inc., Chicago, IL, USA) was used to perform the data analyses. The statistical significance was set for a *P* value of <0.05. The data was tested for normality of distribution using the Shapiro-Wilk test before the test of hypothesis analysis. Mann-Whitney Test was used to analyze the significant difference in age distribution between the test and control group while Chi square test was used to analyze the significant difference in distribution of gender and ethnicity between the test and control group. Independent Samples Test and Mann-Whitney Test were used to analyze the significant difference between the test and control group before and 3 months after the treatment. Paired Sample *t*-test and Wilcoxon Signed Ranks Test were used to analyze the significant difference in the level of HbA1c,* P. gingivalis*,* T. forsythia, *and* A. actinomycetemcomitans *within the same group before and 3 months after the treatment. Repeated Measured ANOVAs were used to analyze the difference in time for each periodontal variable within and between groups.

## 3. Results

The mean age of participants in test and control groups was 37.45 and 44.33 years, respectively, as there was no significant difference between groups in age distribution (*P* > 0.05). For gender distribution, about 40% of the gender in test group were males while the remaining 60% were females. In the control group, 33.3% were males and 66.7% were females. In the test group the distribution of Chinese and Indians participants was equal, 45.0% for each ethnic group, while 10% were Malays and for the control group about 3% were Indians while Malays and Chinese were 42.9% and chi square test detected significant difference in distribution of ethnicity between groups (*P* < 0.05). The majority of participants in the test group had been diabetics for more than 12 years (Table 1).

### 3.1. Clinical Periodontal Response

At the beginning of this study, there was no significant difference in distribution of periodontal parameters between groups.

At 2 months, plaque scores had significantly reduced (*P* < 0.05) from 45.85 ± 27.39 to 14.85 ± 9.37 in test group and from 32.4 ± 20.60 to 13.09 ± 11.3 in the control group. While gingival bleeding score for test group reduced from around 36.70 ± 22.22 at baseline to 19.35 ± 10.81 at 2 months and from 34.04 ± 20.56 basically to 17.52 ± 10.74 at 2 months in the control group. These scores were statistically significant (*P* < 0.05) for both groups. At 3 months of follow-up, both indices remained below 13% in both groups.

PPD < 4 mm at baseline was 79.69 ± 15.31 mm for the test group and 74.96 ± 11.49 for the control group. At 2 months, the mean percentage of sites of PPD < 4 mm increased in the test group and it further increased at the end of 3 months, which was statistically significant (*P* < 0.001). In the control group, the changes from baseline to 2 months and 3 months were also statistically significant (*P* < 0.001).

At 2 months, sites with PPD between 4 and 6 mm reduced by more than 79% in test group and by about 76% in the control group which was statistically significant (*P* < 0.001) in both groups and it further reduced at 3 months which was again statistically significant (*P* < 0.001).

Mean percentage of PPD of >6 mm reduced at 2 and 3 months of follow-up but this reduction was only significant in the control group.

At 2 months and 3 months of follow-up, mean percentage of PAL was significantly reduced in both groups. Comparison between groups was made to detect the difference between test and control groups in the mean percentage of periodontal variable. Statistical tests showed nonsignificant difference between both groups at any point of time.

### 3.2. Metabolic Response

The result of this study showed lowering in the HbA1c value after nonsurgical periodontal therapy in the test group from 9.24 ± 2.34 at baseline to 8.93 ± 2.35 at 3 months of follow-up. However, this reduction was not significant statistically (*P* = 0.111).

### 3.3. Microbiological Response

At baseline, there was no significant difference in the quantity of* P. gingivalis, T. forsythia,* and* A. actinomycetemcomitans *between test and control groups (Table 3).

At the beginning of this study, the quantity of* P. gingivalis *reduced from around 1.4 × 10^7^  ± 2.3 × 10^7^ at baseline to 7.2 × 10^4^  ± 2.7 × 10^4^ at 3 months of follow-up for test group and from 1.8 × 10^7^  ± 2.6 × 10^7^ basically to 3.4 × 10^6^  ± 9.5 × 10^6^ at 3 months for control group which was statistically significant for both groups.

The quantity of* T. forsythia *at baseline was 2.7 × 10^4^  ± 2.0 × 10^4^, 3.0 × 10^4^  ± 2.7 × 10^4^ for test and control groups, respectively. At 3 months, the quantity of this bacterium reduced to 5.2 × 10^3^  ± 1.1 × 10^4^ in test group and to 8.2 × 10^3^  ± 1.6 × 10^4^ in control group. However, the overall reduction was statistically significant in the test and control groups.

The prevalence of* A. actinomycetemcomitans *at baseline for test group and control group was 7.0 × 10^3^  ± 1.2 × 10^4^ and 4.1 × 10^3^  ± 8.5 × 10^3^, respectively; however, this difference between test and control group at baseline was statistically nonsignificant (*P* > 0.738). From baseline to 3 months of follow-up visits both groups showed nonstatistical significant reduction in the mean quantity of* A. actinomycetemcomitans.*

## 4. Discussion

This was a cross-sectional interventional study conducted on Malaysian patients with type 1 diabetes. Most of the researches were conducted locally and in other countries mainly used type 2 patients. Thus this study was one of the first investigations to be conducted on type 1 diabetic Malaysian subjects where the effects of nonsurgical periodontal therapy were evaluated.

In this study there was general improvement of all periodontal parameters in both test and control groups after nonsurgical periodontal therapy with no statistically significant difference between groups at any point of time. The results of this study come in agreement with all previous studies [20–25], which showed no significance clinical difference between diabetic and nondiabetic patients in response to nonsurgical periodontal therapy. Both test and control groups in this study showed more than 65% improvement in plaque scores at 3 months of treatment. Similar findings have been reported by a study conducted by Navarro-Sanchez et al. [22]. As to gingival bleeding scores, both test and control groups showed more than 50% reduction in GBI. Similar finding had been reported in a study conducted by Tervonen et al. [25]. For PPD, there were significant improvements in PPD at the end of 2 months of follow-up in both groups except for PPD of >6 mm for test group. Although there was reduction in mean percentage of sites of PPD of >6 mm, this reduction did not reach significant level statistically. This could be attributed to power of sites of PPD of >6 mm, which was considered too small in the test group (0.370) compared to control group (0.706). However, a deep remaining probing pocket depth of ≥6 mm at reevaluation phase may not indicate a failure of treatment, as around 75% of these sites illustrated improvement of ≥1 mm at reevaluation compared to baseline values [26].

This study also indicated that periodontal therapy did not change the level of HbA1c significantly and this result comes in agreement with previous studies [9, 16, 17, 23, 27]. Conversely, other studies suggest that the control of periodontal infection improves glycemic control [12, 13, 15, 22]. Factors such as periodontal disease severity, HbA1c monitoring duration, type of diabetes, and the use of local or systemic antibiotics could probably explain some of these differences in the results. The addition of antibiotics, usually the tetracycline family to mechanical debridement, has been demonstrated to have a positive effect not only on clinical periodontal and microbiological parameters but also on metabolic control compared to mechanical treatment alone [28]. A pilot study conducted in nine Hispanics in which the mechanical therapy was combined with the systemic administration of doxycycline demonstrated a 0.6% reduction in the level of glycated hemoglobin in type 2 diabetic patients [14]. The other factor which we had to consider was the type of diabetes that the subjects had, as most of studies showed significant effect of periodontal therapy on type 2 diabetes mellitus patients [13, 29], while other studies carried out on type 1 diabetics have shown no significant effect of periodontal therapy on glycemic control [16, 17, 23]. One possible explanation of our results might be because of the small sample size and it was possible that our small sample size prohibited detection of HbAlc changes. However, Janket et al. [30] had suggested that changes in HbAlc might be less evident in type 1 diabetes, as this disease is due to an autoimmune process and is controlled by insulin administration. These patients are known to maintain a tighter control of their glycemic control.

This study showed significant reductions in the quantity of* P. gingivalis* and* T. forsythia *in both groups following nonsurgical periodontal therapy while the level of* A. actinomycetemcomitans* was unaffected, which is in agreement with previous studies [31–33]. Takamatsu et al. [33] investigated the short-term effects of nonsurgical periodontal therapy on the quantity of* P. gingivalis* and* T. forsythia* in 26 periodontally diseased patients using DNA probe. The prevalence of* A. actinomycetemcomitans* was also identified, but, by using PCR, this study demonstrated significant reduction in the levels of* P. gingivalis *and* T. forsythia *while levels of* A. actinomycetemcomitans* were unaffected.

In this study we also found high levels of both* P. gingivalis *and* T. forsythia* in diseased sites before scaling and root planning compared to healthy sites after therapy and the prevalence of* P. gingivalis *and* T. forsythia* decreased significantly after periodontal therapy. The same results have also been reported in other studies [34]. Many studies evaluating the mean percentage of sites colonized by* T. forsythia *in subgingival plaque have demonstrated significantly higher frequency in diseased patients compared to healthy controls [34]. Moreover,* T. forsythia *was closely associated with* P. gingivalis* colonization and was more prevalent in the older age groups [35].

In this study, nonsurgical periodontal therapy did not succeed in reducing the level of* A. actinomycetemcomitans* significantly; similar results have also been reported in the previous studies [36] which reported significant improvements in clinical periodontal parameters combined with reduction in the level of* P. gingivalis *following root debridement, while* A. actinomycetemcomitans *was still present in high proportions. Studies have also demonstrated that it is difficult to eradicate* A. actinomycetemcomitans* from periodontal tissues of aggressive periodontitis by mechanical periodontal therapy only [37], and this could be contributed to the ability of* A. actinomycetemcomitans* to invade deeply into gingival tissue. Furthermore, this study was conducted on subjects with chronic periodontitis, which is mainly caused by red complex species while* A. actinomycetemcomitans* is a microorganism that is highly associated with an aggressive form of periodontal disease found in young adults [38].

Some studies previously also reported that microbiota associated with diabetes does not appear to be different from microbiota of nondiabetics [39]. In this study no significant difference has been reported in the prevalence* P. gingivalis, T. forsythia,* and* A. actinomycetemcomitans* between diabetic and nondiabetic subjects at baseline and 3 months of follow-up, except for* A. actinomycetemcomitans*, which increased in the nondiabetic group at 3 months of follow-up. This could possibly be due to reduced compliance to oral hygiene instructions in several participants in the control group, where higher plaque scores were detected from 2 months to 3 months of follow-up.

In this study, we also found that there were no differences in response to nonsurgical periodontal therapy in clinical parameters, microbiological profile, and glycemic control in the three main ethnic groups (Malay, Chinese, and Indian) found in the Malaysian population.

## 5. Conclusion 

Both test and control treatments produced similar improvements in clinical parameters from baseline to three months after treatment. However, there were no significant differences between test and control groups at any time. The results obtained from this study appear to demonstrate no statistically significant association between clinical improvements in the periodontal condition and improved metabolic control of diabetes. From the available data, it appears that mechanical periodontal therapy is an important aspect in the management of patients with periodontal disease and should be included as the routine protocol in dealing with diabetics.

However, future studies on the association of patients with type 1 diabetes and chronic periodontitis should be conducted on a larger sample size to establish the association and linkage.

## Figures and Tables

**Table 1:** Sociodemographic data of study sample.

	Test group	Control group	*P* value
Age (mean ± SD)	37.45 ± 14.38	44.33 ± 11.48	0.140
Sex			
Female	60%	66.7%	0.658
Male	40%	33.3%	
Race %			
Malay	2 (10%)	9 (42.9%)	0.024
Chinese	9 (45%)	9 (42.9%)	
Indian	9 (45%)	3 (14.3%)	
Duration of diabetes (mean ± SD)			
<7 years	3.12 ± 2.01		
7–12 years	8.80 ± 0.83		
<12 years	29.0 ± 8.83		

*P*: *P* value of comparison between groups.

**Table 2:** Comparison of periodontal parameters between diabetic (test group) and nondiabetic (control group) at baseline and 3 months after periodontal therapy.

	Baseline	2 months	3 months	*P*_1_ value	*P*_2_ value
Plaque score					
Test group	45.85 ± 27.39	14.85 ± 9.37	10.80 ± 10.81	0.000	0.188
Control group	32.4 ± 20.60	13.09 ± 11.3	9.8 ± 5.86	0.000	
Bleeding score					
Test group	36.7 ± 22.22	19.35 ± 10.81	12.15 ± 7.68	0.000	0.671
Control group	34.04 ± 20.56	17.52 ± 10.74	11.76 ± 9.69	0.000	
PPD < 4 mm					
Test group	79.69 ± 15.31	95.73 ± 3.37	97.76 ± 1.87	0.000	0.109
Control group	74.96 ± 11.49	92.69 ± 5.6	95.7 ± 4.13	0.000	
PPD 4–6 mm					
Test group	19.68 ± 13.80	4.07 ± 2.74	2.26 ± 1.82	0.000	0.053
Control group	27.35 ± 13.28	6.44 ± 4.87	4.10 ± 3.62	0.000	
PPD > 6 mm					
Test group	0.68 ± 1.58	0.19 ± 0.74	0.00 ± 0.00	0.065	0.168
Control group	1.78 ± 2.80	0.32 ± 0.73	0.20 ± 0.69	0.006	
PAL < 4 mm					
Test group	7.53 ± 9.98	10.83 ± 13.60	12.34 ± 14.93	0.007	0.150
Control group	9.24 ± 5.5	16.13 ± 8.18	19.56 ± 9.94	0.000	
PAL 4–6 mm					
Test group	26.90 ± 18.85	9.89 ± 11.36	7.46 ± 9.22	0.000	0.075
Control group	34.38 ± 10.69	16.35 ±10.28	11.97 ± 10.02	0.000	
PAL > 6 mm					
Test group	1.85 ± 3.26	0.78 ± 1.64	0.54 ± 1.67	0.012	0.450
Control group	3.12 ± 5.77	1.50 ± 4.15	1.06 ± 3.29	0.004	

*P*_1_: *P* value of overall changes from baseline to 2 months and to 3 months.

*P*_2_: *P* value of comparison between groups at baseline and 3 months.

**Table 3:** Comparison of the quantity of *P. gingivalis*, *T. forsythia*, and *a. Actinomycetemcomitans* between test and control groups at baseline and 3 months of follow-up visits and HbA1c result for test group.

	Baseline	3 months	*P* value
Pg			
Test group	1.4 × 10^7^ ± 2.3 × 10^7^	7.2 × 10^4^ ± 2.7 × 10^4^	0.000
Control group	1.8 × 10^7^ ± 2.6 × 10^7^	3.4 × 10^6^ ± 9.5 × 10^6^	0.033
Tf			
Test group	2.7 × 10^4^ ± 2.0 × 10^4^	5.2 × 10^3^ ± 1.1 × 10^4^	0.000
Control group	3.0 × 10^4^ ± 2.4 × 10^4^	8.2 × 10^3^ ± 1.6 × 10^4^	0.001
Aa			
Test group	7.0 × 10^3^ ± 1.2 × 10^4^	1.3 × 10^3^ ± 4.1 × 10^3^	0.227
Control group	4.1 × 10^3^ ± 8.5 × 10^3^	6.6 × 10^3^ ± 1.4 × 10^4^	0.526
HbA1c %			
Test group	9.24 ± 2.34	8.93 ± 2.35	0.111

P: *P* value of overall changes from baseline to 3 months.
